# Fahr’s Disease Presenting as Mania: A Case Report

**Published:** 2012

**Authors:** Sheikh Shoib, Mohammad Maqbool Dar, Tasleem Arif, Haamid Bashir, Javid Ahmed

**Affiliations:** 1 Department of Psychiatry, GMC Srinagar,; 2 Department of Dermatology, STD and Leprosy, GMC Srinagar,; 3 Department of Biochemistry, GMC Srinagar,; 4 Department Community Medicine, SKIMS Medical College Bemina

**Keywords:** Basal Ganglia, Calcification, Fahr’s Disease, Mania

## Abstract

Fahr’s disease is a rare syndrome characterized by symmetrical and bilateral intracranial calcifications. We report a 21-year-old male who presented with aggression, talkativeness, restlessness, and insomnia of recent onset. His neurological examination was normal. Mental status examination and clinical investigation revealed mania. Brain revealed symmetrical large areas and foci of calcification in bilateral basal ganglia and subcortical regions of cerebral hemispheres. This is the first reported case of mania associated with Fahr’s disease in a Kashmiri patient. The description highlights the importance of considering organic causes when encountering patients with mania. The patient was put on mood stabilizers and his abnormal behaviors improved within 4 weeks.

## Introduction

Fahr’s disease refers to sporadic or familial idiopathic basal ganglia calcification that is associated with many neurological, psychiatric and cognitive abnormalities(1).About 40% of patients with basal ganglia calcification present initially with psychiatric features including cognitive, psychotic, and mood disorders(2).They usually present in the middle age, although an early-onset type mimicking schizophrenia with a more progressive deteriorating course resulting in percentile dementia has also been reported(3).Both sporadic and familial types have been documented in the literature(4).

Mania secondary to idiopathic basal ganglia calcification can occur and has been studied in series of patients. Mood symptoms just resemble those of other disorders affecting subcortical structures (5). Fahr’s disease with bipolar mood disorder presentation is very rare and has been reported (6).The case of a 21-year-old unmarried male withmanic features secondary to Fahr’s disease is presented here.

## Case report

A 21-year-old unmarried Muslim male patientwho was abusinessmanpresented, accompanying by his family members, to our psychiatrydepartment with recent onset of aggressive behavior and over talkativeness. His family members also revealed history of abnormalbehaviorssuch as using abusive language, absconding tendencies, elated mood, spending excess money than usual for daily activities, dressing unusual clothes, and history of grandiose ideas, and stating that he is the king of Kashmir. These abnormal behaviors had started during the last few months before presentation. Recently his sleep pattern has also become quite irregular, sleeping for only two hours a day,and not attending his duties during the last two weeks.There was no family history of psychiatric illness or any neurological illness. His premorbid personalitywas described as even-tempered and hard working. He was neither a smoker nor a drinker and had never abused illicit drugs. He had no past forensic record.

**Figure 1:**
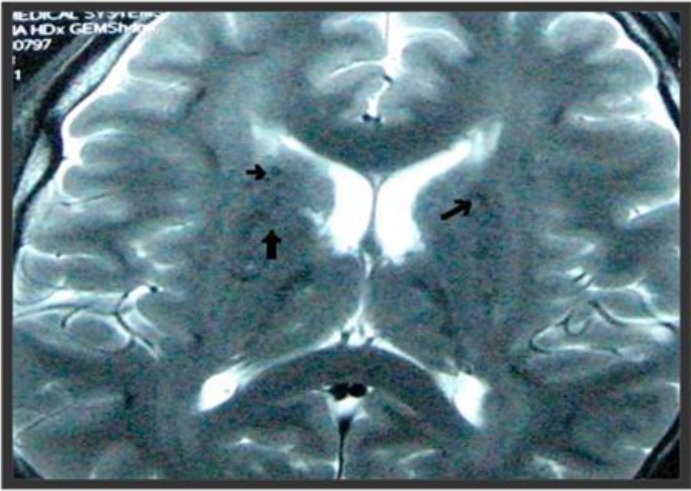
MRI of the brain showing foci of calcification (arrows) in bilateral basal ganglia and subcortical regions of bilateral cerebral hemispheres

 His general physical and neurological examinations were unremarkable. Mental status examination revealedpsychomotor agitation, pressure of speech andelated mood with appropriate affect.Concentration, attention and verbal fluency were also impaired with grandiose ideation.We conducted laboratory investigation for metabolic profile, renal function, liver function as well as workup for inflammatory and infectious conditions, which did not reveal any abnormalities. Bloodinvestigations, including serum calcium and phosphatelevels were normal. His electroencephalogram was normal.Brain MRI showed foci of calcification in bilateral basal ganglia, and subcortical regions of bilateral cerebral hemispheres, thalami and cerebellar parenchyma (Figure 1).

The patient was provisionally diagnosed as bipolar I disorder and was put on mood stabilizer sodium valproate (500 mg twice daily) and olanzapine (10 mg at bedtime). The patient was advised to continue the medication. The symptoms improved dramatically within four weeks of medical therapy initiation. The patient showed marked improvement regarding his abnormal behaviors over the period of three to fourweeks post-treatment.

## Discussion

The typical presentation of Fahr’s disease is movement disorder. The majority of these cases present with Parkinsonian symptoms. Chorea and athetosis have been reported as well. Cognitiveimpairment, speech disorder, and cerebellar symptoms are less common manifestations.

The true prevalence of Fahr’s disease is not known; that of Fahr’s syndrome may be around 0.5%(7).Although Fahr’s disease has generally been considered to be idiopathic, recently a linkage to chromosome 14q in a family with multiple affected members has been described(8).There is no prenatal or genetic test available for genetic counseling of this disease. It has been suggested that tissue damage by free radicals orby abnormal iron transport may trigger calcification(5).The globus pallidus is most affected, but depositions can also be found in the putamen, caudate, thalamus, dentate nucleus, corona radiata, and cerebellar white matter(9).

Diagnosis of Fahr’s disease is made on the basis of clinical features, brain imaging, and exclusion of other causes of intracranial calcification. The neuroimaging findings of symmetric and extensive calcification are usually typical and conspicuous and laid the basis of diagnosis in our case. Other causes of intracranial calcification must be ruled out, such as parathyroid disorders, vascular lesions, infectious diseases, and inflammatory illnesses which were unremarkable in our case.

Our case ofmania associated with Fahr’s disease wassporadic. The patient was managed conservatively. The case illustrates the importance of considering organic causes of mania before labeling them as purely functional and also usefulness of MRI for investigating organic causes of mania and psychosis.

## Authors' contributions

ShSh and MMD conceived, prepared and wrote themanuscript. ShSh and TA participated in the acquisition and interpretation of radiological data .ShSh and JA have been involved in the acquisition of clinical data and in the reviewing the scientific literature. ShSh, TA, HB contributed to the final version and carried out the clinical case report. All authors read and approved the final manuscript**.**

## Notes

**Declaration of interest**: None.
